# Supplementary material for: Germline inherited small RNAs facilitate the clearance of untranslated maternal mRNAs in C. elegans embryos
Source: Nat Commun. 2021 Mar 4;12:1441. doi: 10.1038/s41467-021-21691-6 (PMC7933186; doi:10.1038/s41467-021-21691-6)
Supplement: Supplementary file 1 — Supplementary Information [file 41467_2021_21691_MOESM1_ESM.pdf]

Germline inherited small RNAs facilitate the clearance of untranslated maternal mRNAs in *C. elegans* embryos

Quarato *et al.*

Supplementary Information

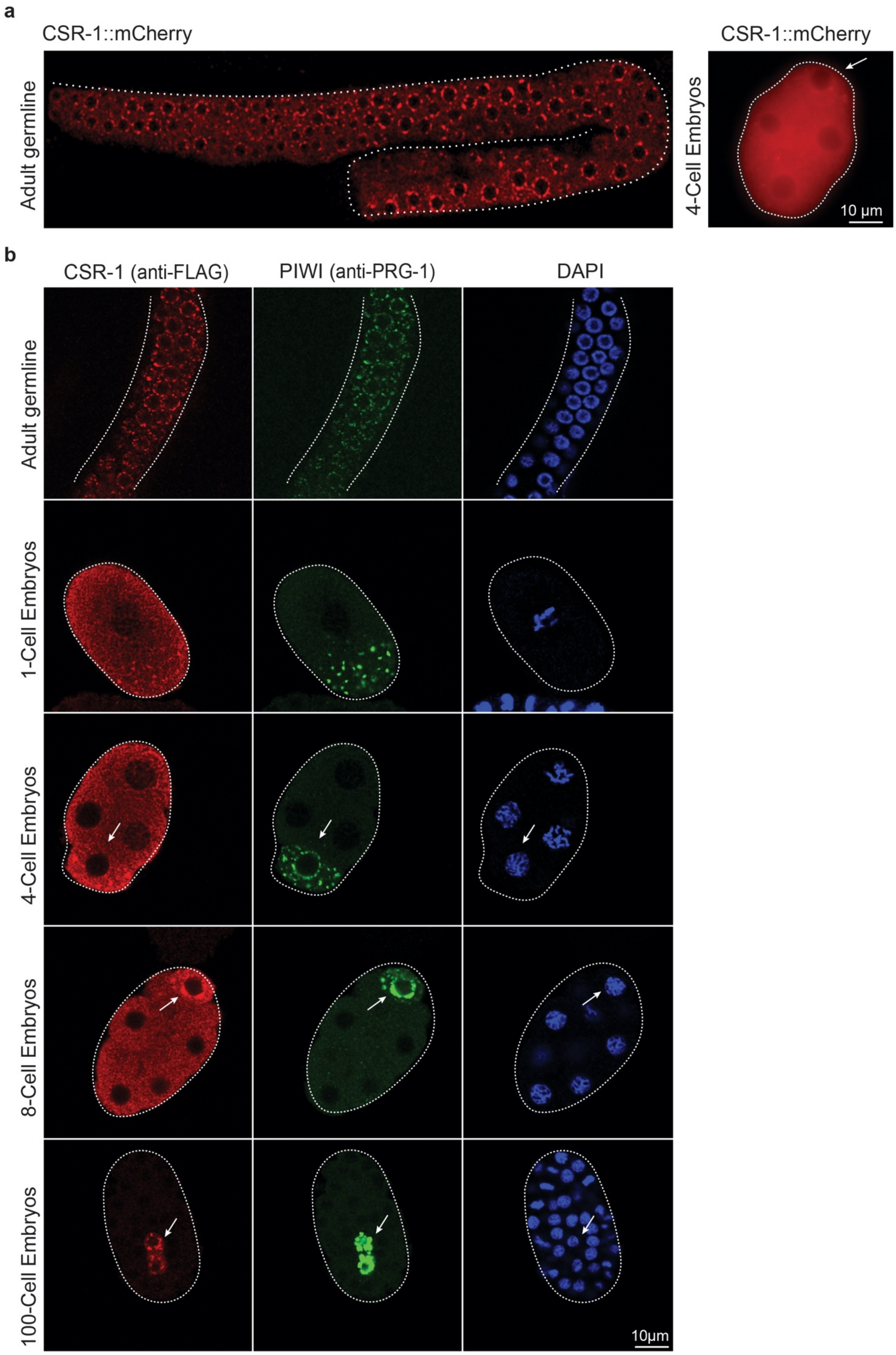
a
CSR-1::mCherry
Adult germline
CSR-1::mCherry
4-Cell Embryos
10 µm
b
CSR-1 (anti-FLAG)
PIWI (anti-PRG-1)
DAPI
Adult germline
1-Cell Embryos
4-Cell Embryos
8-Cell Embryos
100-Cell Embryos
10µm

Supplementary Fig. 1: CSR-1 and PIWI localization in adult germlines and early embryos

a, Confocal image of CSR-1::mCherry in a gonad of late L4 larvae stage (left), and fluorescent micrographs (right) of 4-cell embryo expressing mCherry::3xFLAG::HA::CSR-1. Dashed lines represent gonads and embryos. Scale bars represent 10 μm. Arrows indicate germline blastomeres. **b,** Immunostaining of CSR-1 and PIWI in dissected Adult gonad (top images) and 1-cell, 4-cell, 8-cell, and more than 100-cell embryo (bottom images), as shown in **Fig. 1a,** using anti-FLAG and anti-PRG-1 antibodies. DAPI signal is shown in blue. Dashed lines represent gonads and embryos. Scale bars represent 10 μm. Arrows indicate germline blastomeres. The experiments were repeated
independently two times with similar results.

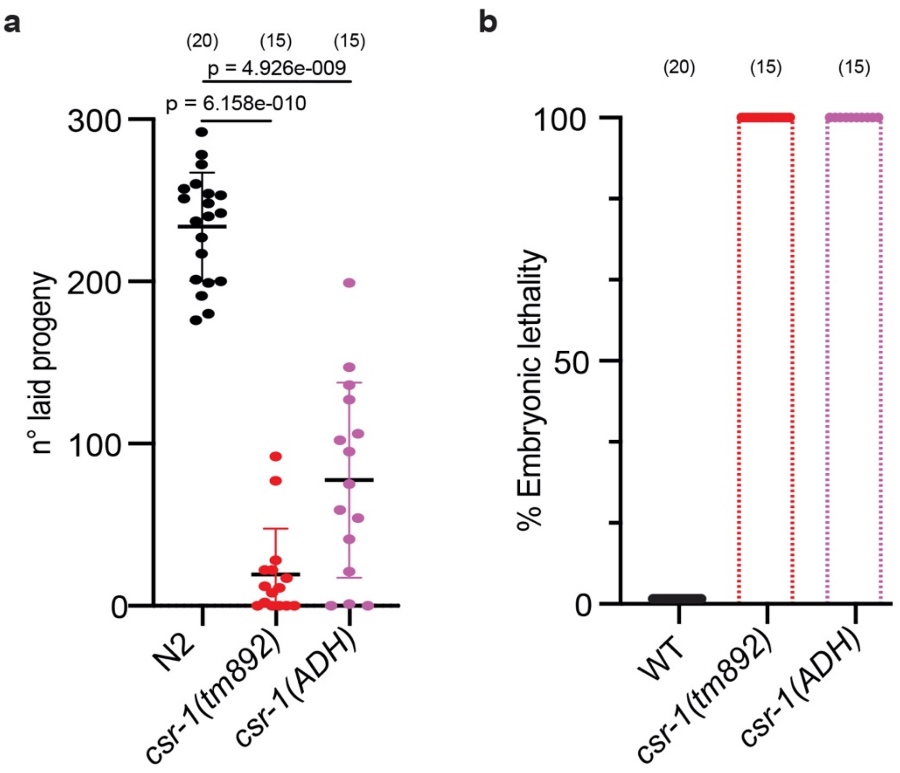

Supplementary Fig. 2: *csr-1(tm892)* mutant and CSR-1 catalytic dead (ADH) show fertility defects and embryonic lethality

a, Brood size assays of *csr-1(tm892)* mutant, CSR-1 catalytic dead (ADH) and wild-type animals. The data points correspond to the number of living larvae from individual worms. Data are presented as mean ± SD. Two-tailed P values were calculated using Mann–Whitney–Wilcoxon tests. **b**, Percentage of embryonic lethality from the brood size experiment shown in **a**, measured as the percentage of dead embryos versus the total number of laid embryos. In **a, b,** the sample size *n* (worms) is indicated in parentheses. Source data are available online.

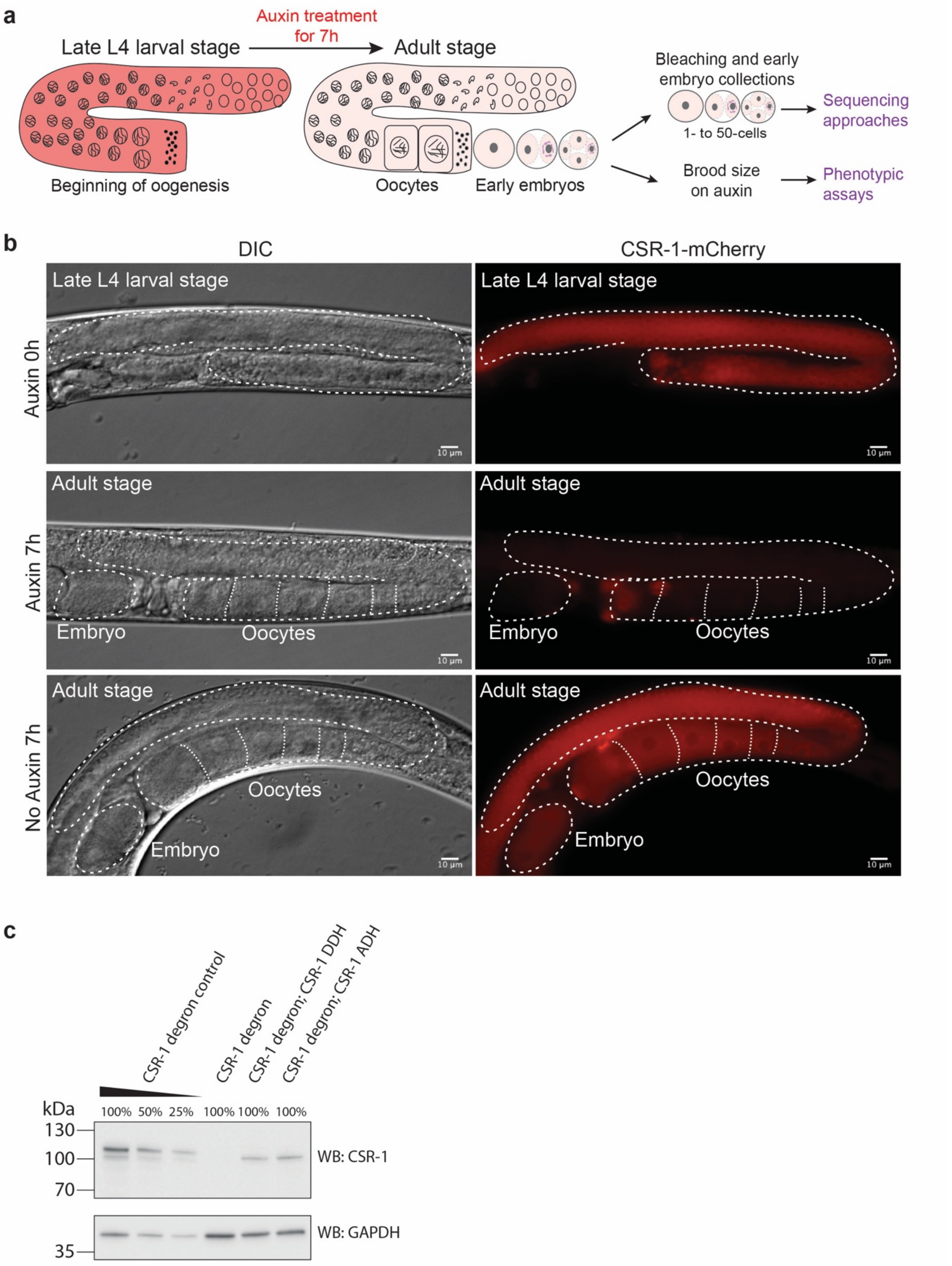
a
Auxin treatment for 7h
Late L4 larval stage
Adult stage
Beginning of oogenesis
Oocytes
Early embryos
Bleaching and early embryo collections
1- to 50-cells
Sequencing approaches
Brood size on auxin
Phenotypic assays
b
DIC
CSR-1-mCherry
Late L4 larval stage
Adult stage
Auxin 0h
Auxin 7h
No Auxin 7h
Embryo
Oocytes
10 µm
c
CSR-1 degron control
CSR-1 degron
CSR-1 degron; CSR-1 DDH
CSR-1 degron; CSR-1 ADH
kDa
100% 50% 25% 100% 100% 100%
130
100
70
35
WB: CSR-1
WB: GAPDH

Supplementary Fig. 3: Depletion of CSR-1 during the MZT using the auxin-inducible degradation (AID) system

a, Schematic of auxin depletion strategy to obtain early embryo populations fully depleted of CSR-1. **b**, DIC micrographs (left) and fluorescent micrographs (right) of live animals expressing Degron::mCherry::3xFLAG::HA::CSR-1 used for auxin depletion experiments (see Materials and methods). Top images show animals before the treatment. Bottom images show gravid animals after treatment with ethanol (no auxin, control) or auxin. Auxin treatment completely depletes CSR-1 in adult gonads and embryos. Dashed lines represent gonads, oocytes and embryos. All scale bars represent 10 μm. The experiment was repeated independently three times with similar results. **c**, Immunoblot showing expression of the CSR-1 protein in worms expressing degron::mCherry::3XFLAG::HA::CSR-1 treated with Ethanol (control, lane 1 to 3) or auxin (lane 4) or expressing degron::mCherry::3XFLAG::HA::CSR-1 and a transgenic copy of catalytic active (DDH) GFP::CSR-1 or catalytic inactive (ADH) GFP::CSR-1 and treated with auxin to deplete the endogenous degron::mCherry::3XFLAG::HA::CSR-1 (lanes 5 and 6). Immunoblot of GAPDH is shown as loading control. The samples derive from the same experiment and blots were processed in parallel. The experiment was repeated independently three times with similar results. Source data are available online.

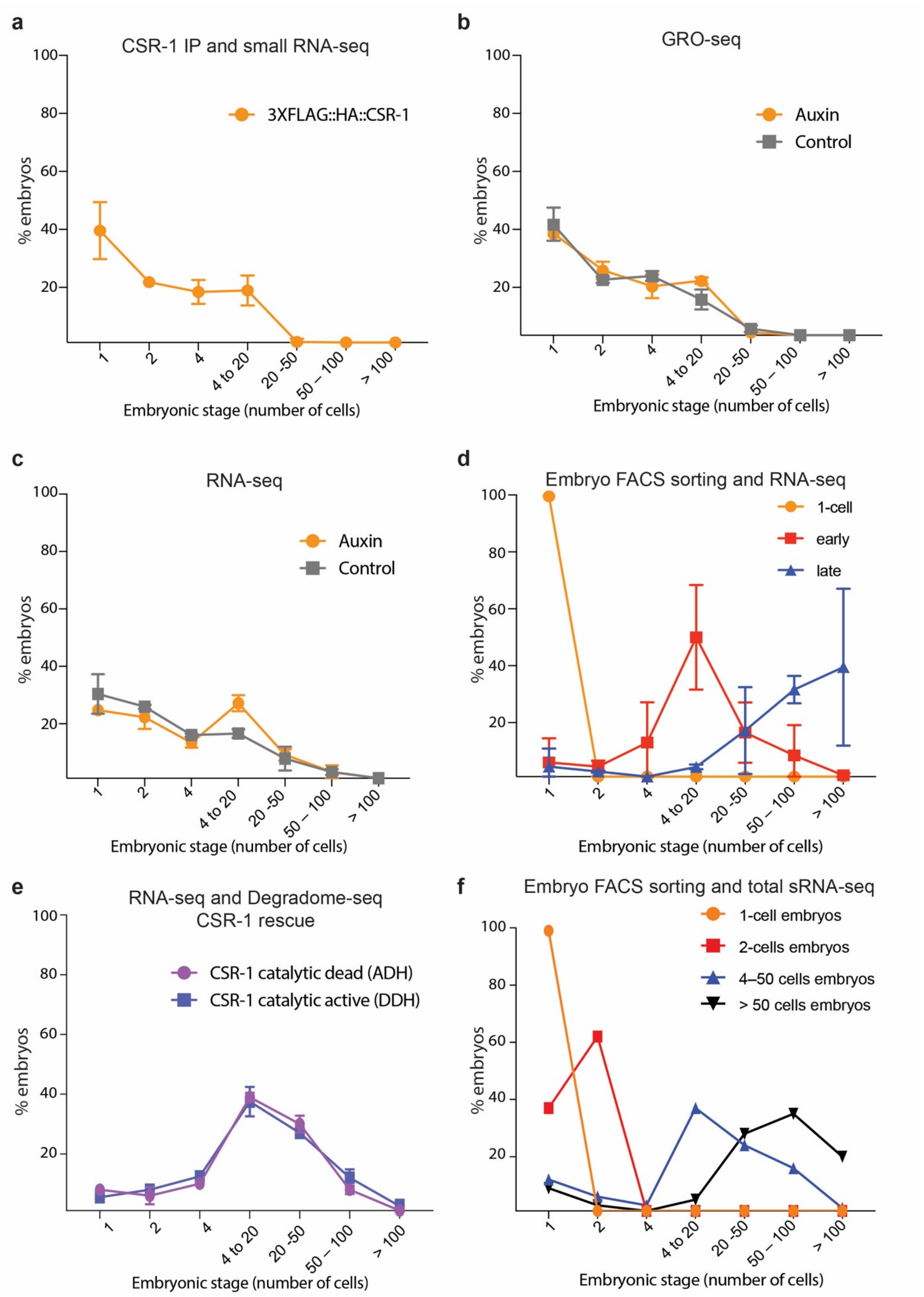
a
CSR-1 IP and small RNA-seq
3XFLAG::HA::CSR-1
% embryos
Embryonic stage (number of cells)
b
GRO-seq
Auxin
Control
% embryos
Embryonic stage (number of cells)
c
RNA-seq
Auxin
Control
% embryos
Embryonic stage (number of cells)
d
Embryo FACS sorting and RNA-seq
1-cell
early
late
% embryos
Embryonic stage (number of cells)
e
RNA-seq and Degradome-seq
CSR-1 rescue
CSR-1 catalytic dead (ADH)
CSR-1 catalytic active (DDH)
% embryos
Embryonic stage (number of cells)
f
Embryo FACS sorting and total sRNA-seq
1-cell embryos
2-cells embryos
4–50 cells embryos
> 50 cells embryos
% embryos
Embryonic stage (number of cells)
1
2
4
4 to 20
20 -50
50 – 100
> 100

Supplementary Fig. 4: Early embryo populations used for sequencing experiments

a-**f**, Composition of embryo populations used for sequencing experiments. Embryos were stained with DAPI, frozen to stop cell divisions, and counted using a fluorescent microscope (see Material and Methods). **a**, 3xFLAG::HA::CSR-1 embryos used for small RNA-seq shown in **Fig. 2a, b**. *n* = 3 biologically independent samples. **b, c,** Degron::mCherry::3xFLAG::HA::CSR-1 embryos treated with auxin or ethanol (No Auxin) used for GRO-seq **b**, and RNA-seq **c**, shown in **Fig. 2c, d,** and **Fig. 3d**. *n* = 3 biologically independent samples for GRO-seq **b,** and *n* = 2 biologically independent samples for RNA-seq **c**. **d**, Embryos expressing OMA-1::mCherry; PIE-1::GFP embryos used for sorting 1-cell, early and late embryo populations for RNA-seq shown in **Fig. 3a-c**. *n* = 2 biologically independent samples. **e**, CSR-1 depleted early embryos rescued with transgenic expression of CSR-1 ADH or CSR-1 DDH used for RNA-seq and Degradome-seq shown in **Fig. 4a, c**. *n* = 2 biologically independent samples. **f**, Embryos expressing OMA-1::mCherry; PIE-1::GFP embryos used for sorting 1-cell, 2-cells, 4-50 cells, >50 cells embryo populations for small RNA-seq shown in **Fig. 3e**. *n* = 1 sample. In all graphs data are presented as mean ± SD. Source data are available online.

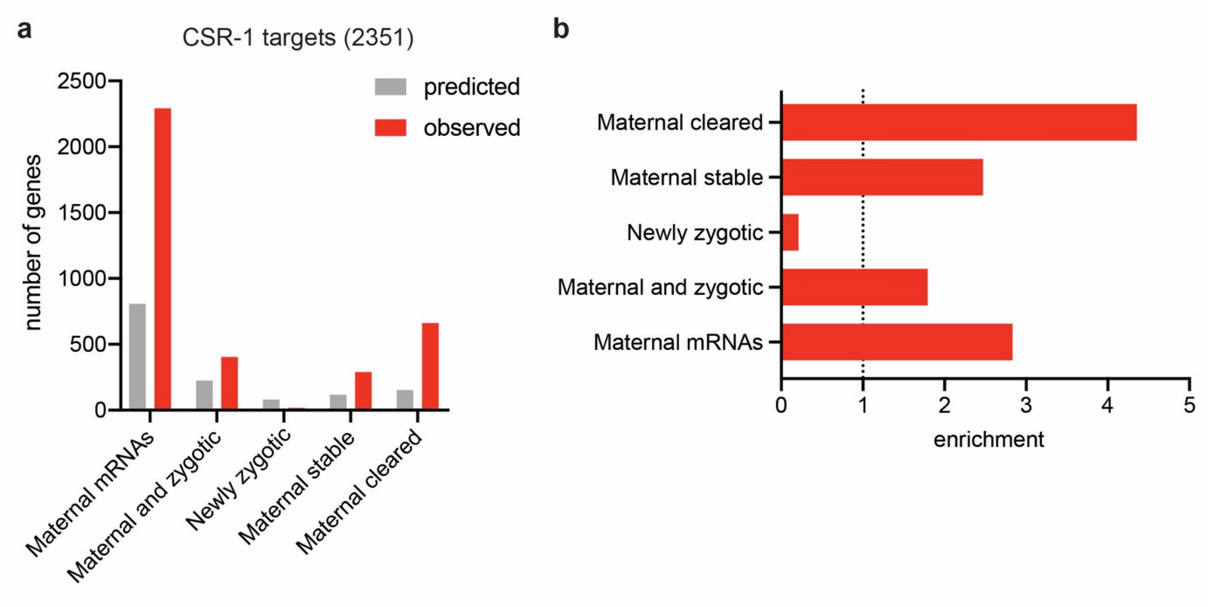

Supplementary Fig. 5: CSR-1 embryonic targets are enriched for maternal degraded mRNAs

a, b, Predicted (grey) and observed (red) distribution (**a**) and enrichment (**b)** of CSR-1 embryonic targets (>1 RPM) in different gene expression categories. Dashed line at 1 indicate no enrichment. Source data are available online.

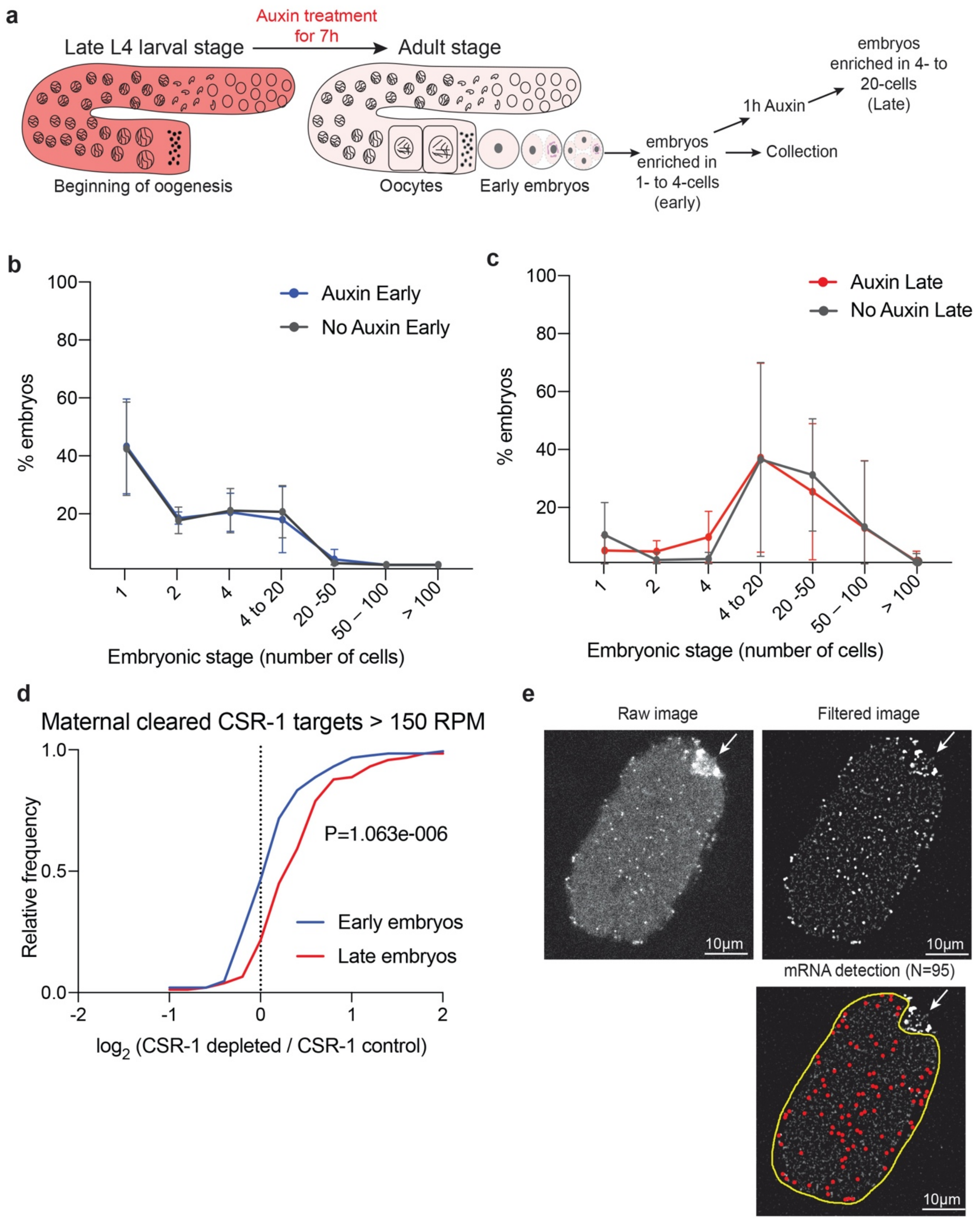

Supplementary Fig. 6: The degradation of maternal mRNAs by CSR-1 occurs in early embryos

a, Schematic of strategy to collect embryo preparations at different stages fully depleted of CSR-1. **b**, **c**, Degron::mCherry::3xFLAG::HA::CSR-1 embryos treated with auxin or control (No Auxin) of early **b**, and late embryos **c**, used for the RNA-seq shown in **d**. *n* = 3 biologically

independent samples. In all graphs data are presented as mean ± SD. **d**, Cumulative distribution of the log_2 fold change of maternal cleared mRNA targets (> 150 RPM) in CSR-1 depleted early embryos compared to CSR-1 depleted late embryos. Sample size n = 661. Two-tailed P values were calculated using Mann–Whitney–Wilcoxon tests. **e**, Example of quantified image by smFISH of *C01G8.1* mRNA target taken in the central plane of embryos at 20-cells stage. Only somatic cell areas were quantified and germline blastomere were excluded from the quantification. Arrow indicates germline blastomere mRNAs. DAPI staining was used to count number of cells per embryo. The number of embryos used for quantification is indicated in parentheses. Scale bars represent 10 μm. The experiment was repeated independently three times with similar results. Source data are available online.

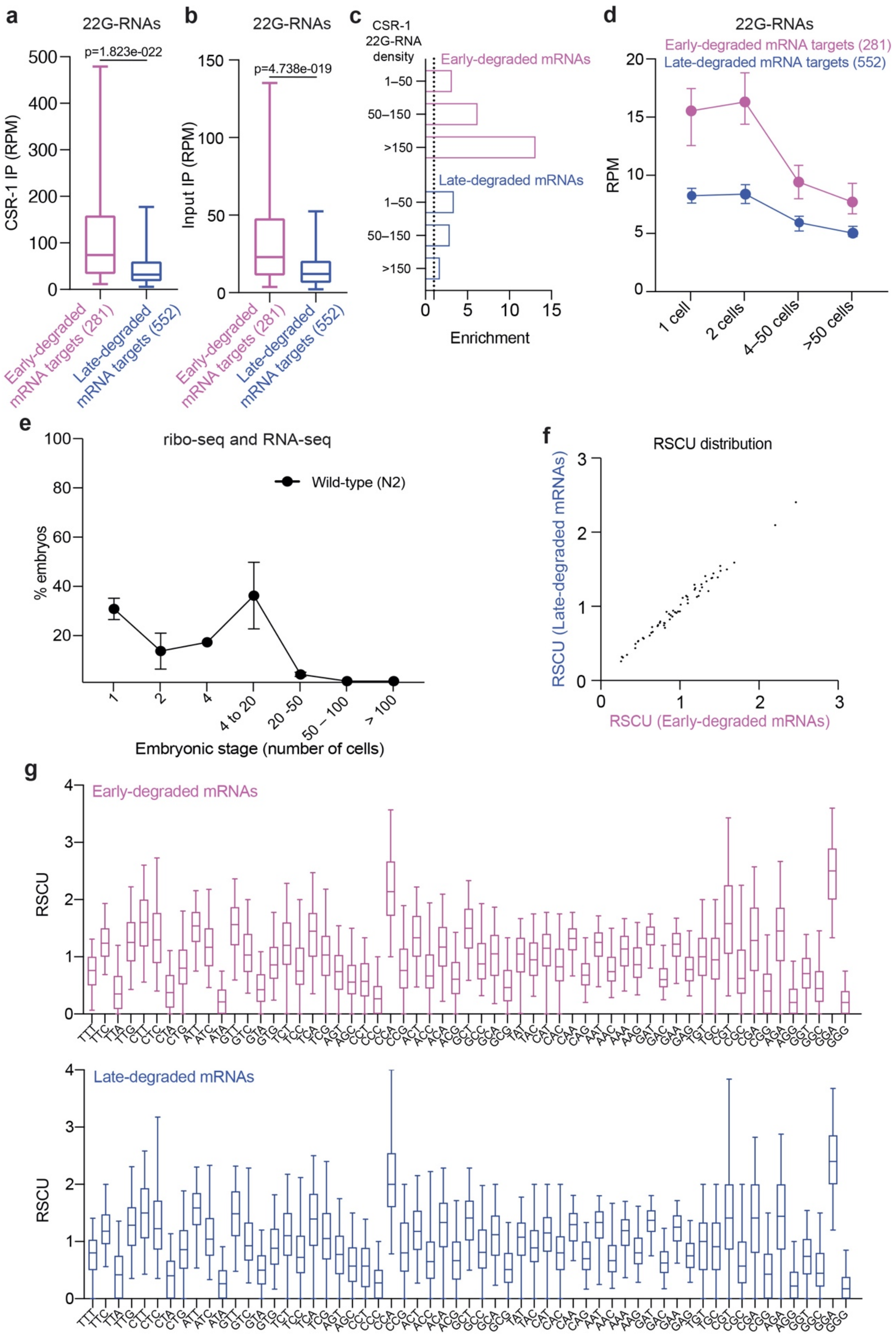

a
22G-RNAs
p=1.823e-022
CSR-1 IP (RPM)
Early-degraded mRNA targets (281)
Late-degraded mRNA targets (552)
b
22G-RNAs
p=4.738e-019
Input IP (RPM)
Early-degraded mRNA targets (281)
Late-degraded mRNA targets (552)
c
CSR-1 22G-RNA density
Early-degraded mRNAs
Late-degraded mRNAs
1–50
50–150
>150
Enrichment
d
22G-RNAs
Early-degraded mRNA targets (281)
Late-degraded mRNA targets (552)
RPM
1 cell
2 cells
4–50 cells
>50 cells
e
ribo-seq and RNA-seq
Wild-type (N2)
% embryos
Embryonic stage (number of cells)
f
RSCU distribution
RSCU (Late-degraded mRNAs)
RSCU (Early-degraded mRNAs)
g
Early-degraded mRNAs
Late-degraded mRNAs
RSCU

Supplementary Fig. 7: CSR-1 preferentially targets early-degraded mRNAs

a, **b**, Box plots showing the abundance of CSR-1-bound 22G-RNAs (**a**) or total input 22G-RNAs (**b**) antisense to early- and late-degraded mRNAs. The line indicates the median value, the box indicates the first and third quartiles, and the whiskers indicate the 5th and 95th percentiles, excluding outliers. Two-tailed p value were calculated using the Mann-Whitney-Wilcoxon test. The sample size *n* (genes) is indicated in parentheses. **c**, Enrichment of CSR-1 embryonic targets in early- or late-degraded mRNAs for gene sets of increasing 22G RNA density, 1–50 RPM, 50–150 RPM, > 150 RPM or non-target genes. Dashed line at 1 indicate no enrichment. **d**, 22G-RNAs levels from sorted embryos enriched in 1-cell stage, 2-cell stage, 4-50 cell stage, 50 cell stage. Data are presented as median and 95% confident interval of normalized 22G-RNA read abundances in transcript per million (TPM) from early- or late-degraded mRNA targets. The sample size used *n* (genes) is indicated in parentheses. **e**, Wild-type embryos used for Ribo-seq and RNA-seq in **Fig. 5e**, **f**. Data are presented as mean ± SD. *n* = 2 biologically independent samples. **f**, Average of relative synonymous codon usage (RSCU) for each codon in early- or late-degraded mRNAs. **g**, Box plots showing the RSCU of early- (top) or late-degraded mRNAs (bottom). The line indicates the median value, the box indicates the first and third quartiles, and the whiskers indicate the 5th and 95th percentiles, excluding outliers. The sample size used to derive statistics is *n (*Early-degraded mRNAs) = 1260 and *n (*Late-degraded mRNAs) = 2769. Source data are available online.

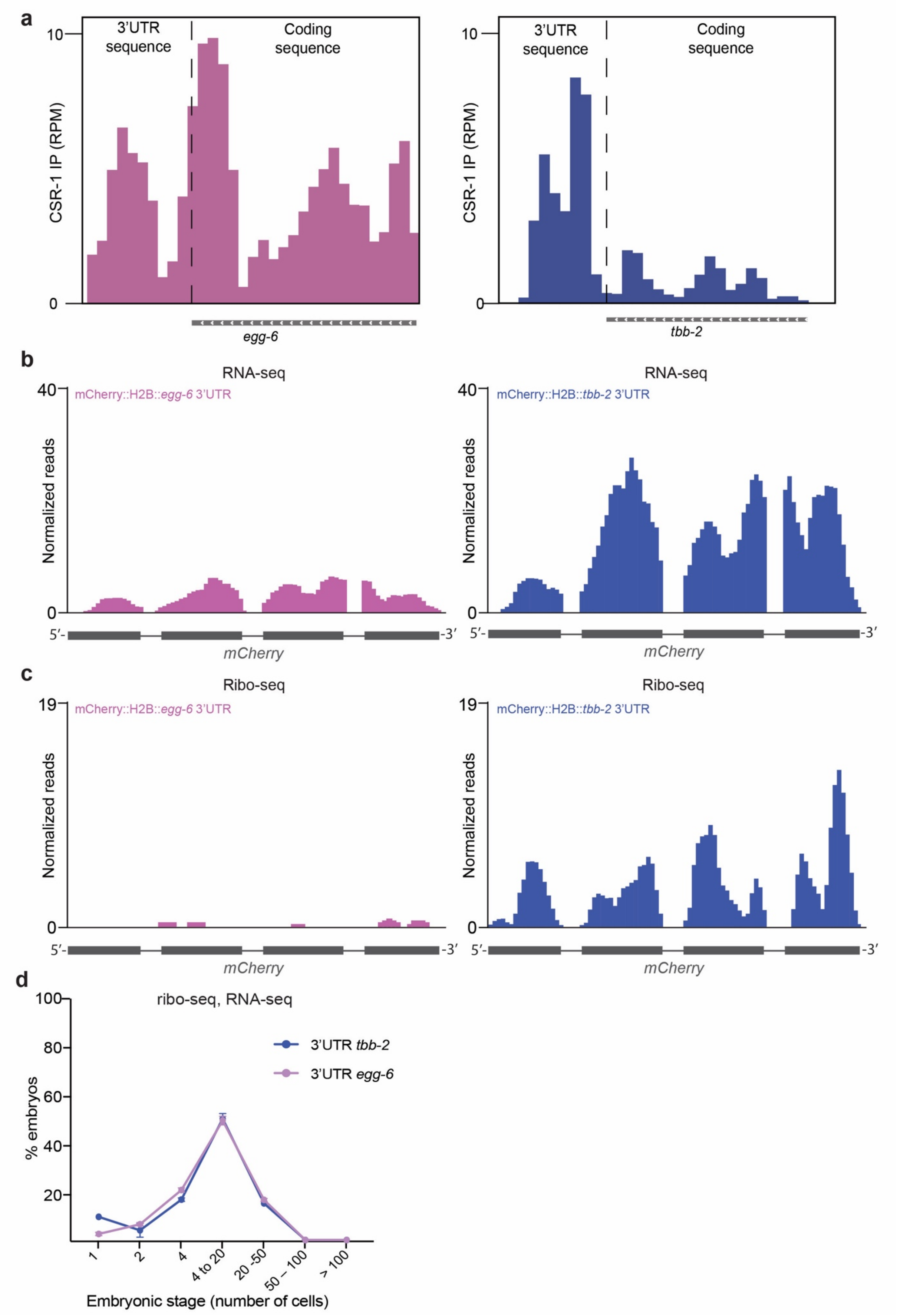
a
3'UTR sequence
Coding sequence
CSR-1 IP (RPM)
10
0
egg-6
tbb-2
b
RNA-seq
mCherry::H2B::egg-6 3'UTR
mCherry::H2B::tbb-2 3'UTR
Normalized reads
40
0
5'-
-3'
mCherry
c
Ribo-seq
19
d
ribo-seq, RNA-seq
3'UTR tbb-2
3'UTR egg-6
% embryos
100
80
60
40
20
1
2
4
4 to 20
20 -50
50 – 100
> 100
Embryonic stage (number of cells)

Supplementary Fig. 8: 3'UTRs derived from early- or late-degraded mRNAs affects translation and mRNA decay in embryos

a, Genomic view of normalized CSR-1-bound 22G-RNAs antisense to the endogenous *tbb-2* and *egg-6* mRNAs. The grey box indicates the last exon and the dashed line delineates the end of the coding sequence and the beginning of 3'UTR sequence. The average from two biologically independent replicates is shown. **b**, **c**, Genomic view of normalized reads from RNA-seq or Ribo-seq on *mCherry* coding sequence in embryos expressing *mCherry::h2b* transgene fused to 3'UTR derived from early- (*egg-6 3'UTR*) or late-degraded (*tbb-2 3'UTR*) mRNAs. The average from two biologically independent replicates is shown. **d**, Embryo populations expressing one of the two single-copy transgenic reporters *mCherry::h2b* mRNA fused to egg-6 3'UTR (early-degraded mRNAs) or tbb-2 3'UTR (late-degraded mRNAs) that have been used for Ribo-seq and RNA-seq in **Fig. 6c**. data are presented as mean ± SD. $n = 2$ biologically independent samples. Source data are available online.

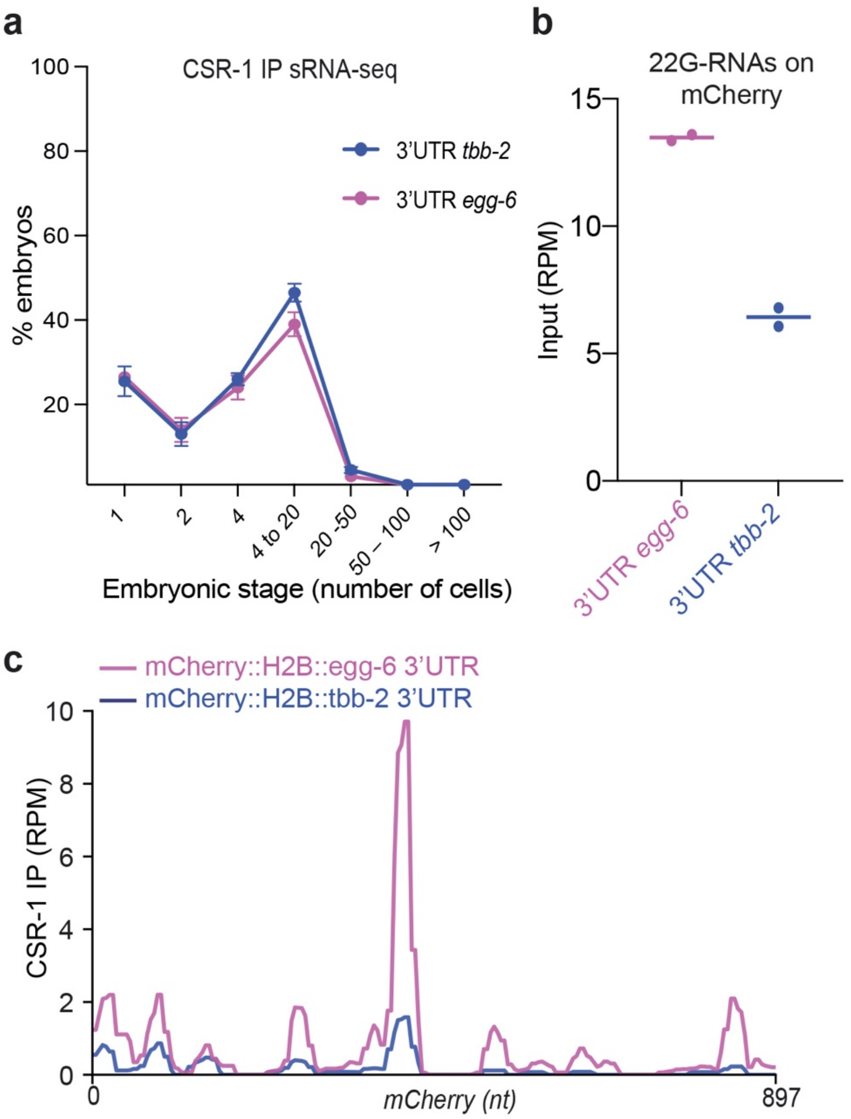

Supplementary Fig. 9: 3'UTRs derived from early- or late-degraded mRNAs affects the levels of CSR-1-bound 22G-RNAs

a, Embryo populations expressing one of the two single-copy transgenic reporters *mCherry::h2b* mRNA fused to egg-6 3'UTR (early-degraded mRNAs) or tbb-2 3'UTR (late-degraded mRNAs) that have been used for CSR-1 IP sRNA-seq in **Fig. 6d, e.** data are presented as mean ± SD. $n = 2$ biologically independent samples. **b**, Abundance of total Input 22G-RNAs antisense to mCherry (in embryos expressing *mCherry::h2b* transgene fused to 3'UTR derived from early- (*egg-6 3'UTR*) or late-degraded (*tbb-2 3'UTR*) mRNAs). The lines indicate the mean and the dots individual data from two biologically independent experiments (left). **c**,

Metaprofile analysis showing normalized 22G-RNA reads (RPM) across *mCherry* nucleotide sequence from *mCherry::h2b* transgenic mRNA fused to *egg-6 3'UTR* (purple) or *tbb-2 3'UTR* (blue) in CSR-1 immunoprecipitation (IP). The average from two biologically independent replicates is shown. Source data are available online.
