# Supplementary material for: Germline inherited small RNAs facilitate the clearance of untranslated maternal mRNAs in C. elegans embryos
Source: Nat Commun. 2021 Mar 4;12:1441. doi: 10.1038/s41467-021-21691-6 (PMC7933186; doi:10.1038/s41467-021-21691-6)
Supplement: Supplementary file 8 — Description of Additional Supplementary Files [file 41467_2021_21691_MOESM8_ESM.pdf]

Description of Additional Supplementary Files

File: Supplementary Data 1
Description: Gene lists generated and used in this study and associated TPM values.

File: Supplementary Data 2
Description: Strain lists generated and/or used in this study.

File: Supplementary Data 3
Description: CRISPR-Cas9 guide RNA lists used in this study.

File: Supplementary Data 4
Description: Primer lists used for RT-qPCR.

File: Supplementary Data 5
Description: Oligos used for smFISH.
