# Supplementary material for: Germline inherited small RNAs facilitate the clearance of untranslated maternal mRNAs in C. elegans embryos
Source: Nat Commun. 2021 Mar 4;12:1441. doi: 10.1038/s41467-021-21691-6 (PMC7933186; doi:10.1038/s41467-021-21691-6)
Supplement: Supplementary file 9 — Reporting Summary [file 41467_2021_21691_MOESM9_ESM.pdf]

natureresearch

Corresponding author(s): Germano Cecere

Last updated by author(s): Jan 18, 2021

Reporting Summary

Nature Research wishes to improve the reproducibility of the work that we publish. This form provides structure for consistency and transparency in reporting. For further information on Nature Research policies, see Authors & Referees and the Editorial Policy Checklist.

Statistics

For all statistical analyses, confirm that the following items are present in the figure legend, table legend, main text, or Methods section.

n/a	Confirmed	
☐	☒	The exact sample size (*n*) for each experimental group/condition, given as a discrete number and unit of measurement
☐	☒	A statement on whether measurements were taken from distinct samples or whether the same sample was measured repeatedly
☐	☒	The statistical test(s) used AND whether they are one- or two-sided<br>*Only common tests should be described solely by name; describe more complex techniques in the Methods section.*
☒	☐	A description of all covariates tested
☒	☐	A description of any assumptions or corrections, such as tests of normality and adjustment for multiple comparisons
☐	☒	A full description of the statistical parameters including central tendency (e.g. means) or other basic estimates (e.g. regression coefficient) AND variation (e.g. standard deviation) or associated estimates of uncertainty (e.g. confidence intervals)
☐	☒	For null hypothesis testing, the test statistic (e.g. *F*, *t*, *r*) with confidence intervals, effect sizes, degrees of freedom and *P* value noted<br>*Give P values as exact values whenever suitable.*
☒	☐	For Bayesian analysis, information on the choice of priors and Markov chain Monte Carlo settings
☒	☐	For hierarchical and complex designs, identification of the appropriate level for tests and full reporting of outcomes
☒	☐	Estimates of effect sizes (e.g. Cohen's *d*, Pearson's *r*), indicating how they were calculated

Our web collection on statistics for biologists contains articles on many of the points above.

Software and code

Policy information about availability of computer code

Data collection

Applied Biosystems™ QuantStudio™ 3 Real-Time PCR System was used for qPCR

Illumina NextSeq 500 or MiniSeq platforms were used for sequencing

Confocal images were taken using ZEISS LSM 700 microscope with 40X objective or 63X objective. Images were obtained using ZEISS ZEN black 2.3 SP1.

Data analysis

For all the sequencing analyses:
- Demultiplexing with Illumina bcl2fastq converter version v2.17.1.14
- Quality control with fastQC version v0.11.5

For RNA-seq analyses:
- Allignment with HISAT2 version 2.0.4
- Genomic features annotation with featureCounts version 1.5.2
- Differential gene expression analysis with DESeq2 version 1.20.0

Small RNA-seq, GRO-seq, Ribo-seq, degradome-seq analyses:
- Demultiplexing with Illumina bcl2fastq converter version v2.17.1.14
- Quality control with fastQC version v0.11.5
- 3' adaptor trimming with Cutadapt version 1.15
- nucleotide size selection using bioawk (https://github.com/lh3/bioawk)
- Allignment with Bowtie2 version 2.3.4.1
- Metaprofile with deepTools package (version 3.4.3)
- Bigwig files with bamCoverage from deepTools (version 3.4.3).

CRISPR-Cas9:
Unique and specific guide RNA sequences were selected using the off-target prediction CRISPR Design tool at http://crispr.mit.edu/.
Quantification of confocal images was performed using ImageJ software V2.0.0.

qPCR data were analyzed using QuantStudioTM Design and Analysis software V 2.2

For manuscripts utilizing custom algorithms or software that are central to the research but not yet described in published literature, software must be made available to editors/reviewers. We strongly encourage code deposition in a community repository (e.g. GitHub). See the Nature Research guidelines for submitting code & software for further information.

Data

Policy information about availability of data

All manuscripts must include a data availability statement. This statement should provide the following information, where applicable:
- Accession codes, unique identifiers, or web links for publicly available datasets
- A list of figures that have associated raw data
- A description of any restrictions on data availability

All the sequencing data are available at the following accession numbers GSE146062. Custom bash script used for sequencing analyses are available at. All other data supporting the findings of this study are available from the corresponding author on reasonable request.

Field-specific reporting

Please select the one below that is the best fit for your research. If you are not sure, read the appropriate sections before making your selection.

☒ Life sciences ☐ Behavioural & social sciences ☐ Ecological, evolutionary & environmental sciences

For a reference copy of the document with all sections, see nature.com/documents/nr-reporting-summary-flat.pdf

Life sciences study design

All studies must disclose on these points even when the disclosure is negative.

Sample size	In all the sequencing experiments at least 40,000 embryos or Adult worms were used. This sample size has been empirically evaluated to be sufficient to obtain large amount of RNAs, sufficient for multiple applications.<br>Sample size for the brood size experiments was determined according to our pre-tests in the lab using at least 10 worms for each strain.
Data exclusions	Dead worms were excluded from the counting in brood size experiments.
Replication	Almost all the experiments shown in this study were performed independently at least two times and no inconsistent results were observed. All attempts at replication were successful.
Randomization	Our experiments were not randomized and controlling covariates was not necessary. For all experiments, control vs. experimental samples were treated in parallel. Samples were allocated into different groups based on genotype for all genetic/mutant analyses performed. Animals were randomly chosen from stock plates for brood size experiments.
Blinding	Plates for brood size assays were assigned with randomized labels and the investigators counting each group were blinded to each group identity.

Reporting for specific materials, systems and methods

We require information from authors about some types of materials, experimental systems and methods used in many studies. Here, indicate whether each material, system or method listed is relevant to your study. If you are not sure if a list item applies to your research, read the appropriate section before selecting a response.

Materials & experimental systems

n/a	Involved in the study	
☐	☒	Antibodies
☒	☐	Eukaryotic cell lines
☒	☐	Palaeontology
☐	☒	Animals and other organisms
☒	☐	Human research participants
☒	☐	Clinical data

Methods

n/a	Involved in the study	
☒	☐	ChIP-seq
☒	☐	Flow cytometry
☒	☐	MRI-based neuroimaging

Antibodies

Antibodies used	The antibody used were:

anti-PRG-1 antibody, a gift from the Craig Mello lab (Claycomb et al., Cell 2009), dilution used for immunistaining 1:800
anti-CSR-1 antibody a gift from the Claycomb Lab (Batista et al. Mol. Cell 2008), diluition used for western blotting 1:3000
anti-FLAG antibody (F3165, Sigma) dilution used for immunostaining 1:500
anti-GAPDH (Ab125247, Abcam), dilution used for western blotting 1:2000
HRP conjugated anti-rabbit (31460, Pierce) dilution used for western blotting 1:10000
HRP conjugated anti-mouse (31430, Pierce) dilution used for western blotting 1:10000
Goat anti-mouse Alexa Fluor 488 (Invitrogen, A11001) dilution for immunostaining 1:500
Goat anti-rabbit Alexa Fluor 568 (Invitrogen A11011) dilution for immunostaining 1:500

Validation

anti-PRG-1 antibody was validated in Batista et al. Mol. Cell 2008.
anti-CSR-1 was validated in Claycomb et al., Cell 2009.
anti-FLAG antibody (F3165, Sigma) has been validated by vendor (https://www.sigmaaldrich.com/catalog/product/sigma/f3165).
anti-GAPDH (Ab125247, Abcam) has been validated by Barucci et al. Nature Cell Biology 2020.
HRP conjugated anti-rabbit (31460, Pierce) has been validated by Barucci et al. Nature Cell Biology 2020.
HRP conjugated anti-mouse (31430, Pierce) has been validated by Barucci et al. Nature Cell Biology 2020.
Goat anti-mouse Alexa Fluor 488 (Invitrogen, A11001) has been validated by vendor (https://www.thermofisher.com/antibody/product/Goat-anti-Mouse-IgG-H-L-Cross-Adsorbed-Secondary-Antibody-Polyclonal/A-11001).
Goat anti-rabbit Alexa Fluor 568 (Invitrogen A11011) has been validated by vendor (https://www.thermofisher.com/antibody/product/Goat-anti-Rabbit-IgG-H-L-Cross-Adsorbed-Secondary-Antibody-Polyclonal/A-11011).

Animals and other organisms

Policy information about studies involving animals; ARRIVE guidelines recommended for reporting animal research

Laboratory animals

All the data collected for this study derived from Adults or embryos hermaphrodite Caenorhabditis elegans strains listed below:
N2, C. elegans wild isolate (Bristol)
MHE27, csr-1(gc017[csr-1::3xFLAG::1HA])IV
MHE54, csr-1(gc027[mCherry::3xflag::1ha::csr-1])IV
MHE69, ieSi64 [gld-1p::TIR1::mRuby::gld-1 3'UTR + Cbr-unc-119(+)] II; csr-1(gc029[degron::mCherry::3xflag::ha::csr-1])
MHE113, ieSi64 [gld-1p::TIR1::mRuby::gld-1 3'UTR + Cbr-unc-119(+)] II; csr-1(gc029[degron::mCherry::3xflag::ha::csr-1]) ; gcSi1 [mex-5p::csr1[D769A]::GFP::tbb-2 3'UTR + Cbr-unc-119(+)] IV
MHE114, ieSi64 [gld-1p::TIR1::mRuby::gld-1 3'UTR + Cbr-unc-119(+)] II; csr-1(gc029[degron::mCherry::3xflag::ha::csr-1]) ; gcSi1 [mex-5p ::csr1::GFP::tbb-2 3'UTR + Cbr-unc-119(+)] IV
MHE34, oma-1(gc013[mcherry::oma-1]) IV; pie-1(ne4301[pie-1::GFP])
JDU128, ijmSi31 [mex-5p::mCherry::his11:: tbb-2 3'UTR]II; unc-119(ed3)III.
MHE121, ijmSi31 [mex-5p::mCherry::his11:: egg-6 3'UTR]II; unc-119(ed3)III.
MHE20, csr-1(tm892)/nT1 [unc-?(n754) let-? qIs51] (IV;V)
MHE21, csr-1(gc010)[D769A]/nT1 [unc-?(n754) let-? qIs51] (IV;V)

Wild animals

No wild animals has been used in this study

Field-collected samples

This study did not involve samples collected from the field.

Ethics oversight

No ethical approval was required.

Note that full information on the approval of the study protocol must also be provided in the manuscript.
