# Supplementary figures and images for: Germline inherited small RNAs facilitate the clearance of untranslated maternal mRNAs in C. elegans embryos
Source: Nat Commun. 2021 Mar 4;12:1441. doi: 10.1038/s41467-021-21691-6 (PMC7933186; doi:10.1038/s41467-021-21691-6)

Uncropped Blots shown in Extended Data Fig. 3c

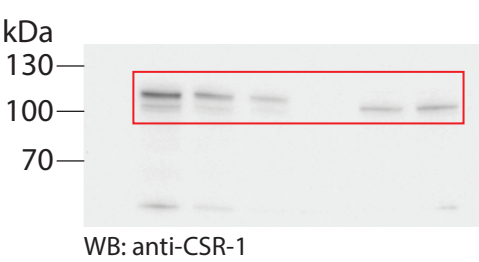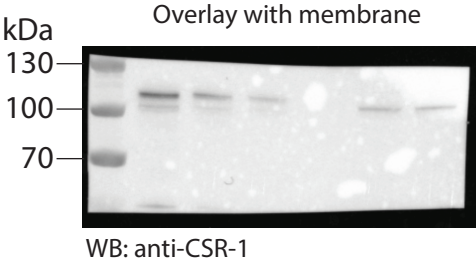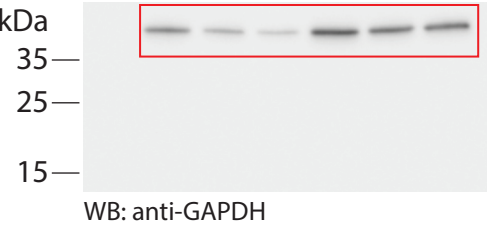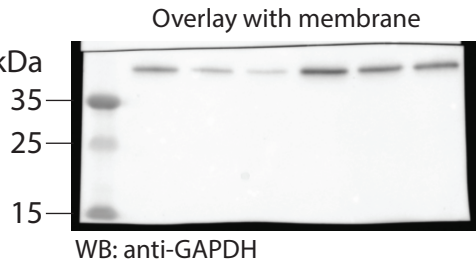

Supplement: Supplementary file 10 — Source Data [file 41467_2021_21691_MOESM10_ESM.zip › Source Data Extended Data Fig3c.pdf]
